# Case report: Is severe toxicity the price to pay for high sensitivity to checkpoint inhibitors immunotherapy in desmoplastic melanoma?

**DOI:** 10.3389/fimmu.2024.1369531

**Published:** 2024-05-10

**Authors:** Teresa Squicciarini, Rossella Villani, Benedetta Apollonio, Livia Fucci, Milena Zambetti, Michele Rossini, Rosamaria Pinto, Stefania Tommasi, Ileana De Roma, Sabino Strippoli, Michele Guida

**Affiliations:** ^1^ Rare Tumors and Melanoma Unit, IRCCS Istituto Tumori Giovanni Paolo II, Bari, Italy; ^2^ Pathology Unit, IRCCS Istituto Tumori Giovanni Paolo II, Bari, Italy; ^3^ Nephrology, Dialysis and Transplantation, Department of Precision and Regenerative Medicine and Ionian Area, University of Bari, Bari, Italy; ^4^ Molecular Diagnostics and Pharmacogenetics Unit, IRCCS Istituto Tumouri “Giovanni Paolo II”, Bari, Italy

**Keywords:** desmoplastic melanoma, checkpoint immunotherapy, renal toxicity, case report, irAE, multi-organ toxicity

## Abstract

**Background:**

Desmoplastic melanoma (DM) is a rare subtype of melanoma characterized by high immunogenicity which makes it particularly suitable for immune checkpoint inhibitors (ICIs) treatment.

**Case presentation:**

We report the case of a 53-year-old man with metastatic DM successfully treated with the combination of anti-CTLA-4 and anti-PD-1 antibodies, who developed serious immune-related adverse events (irAEs). The primary tumor was characterized by absent PD-L1 expression and no-brisk lymphocytes infiltration. NGS showed absence of BRAF mutation, a high tumor mutational burden, and an UV-induced DNA damage signature. Metastatic lesions regressed rapidly after few cycles of ICIs until complete response, however the patient developed serious irAEs including hypothyroidism, adrenal deficiency, and acute interstitial nephritis which led to the definitive suspension of treatment. Currently, the patient has normal renal functionality and no disease relapse after 26 months from starting immunotherapy, and after 9 months from its definitive suspension.

**Conclusion:**

Efficacy and toxicity are two sides of the same coin of high sensitivity to ICIs in DM. For this reason, these patients should be closely monitored during ICIs therapy to promptly identify serious side effects and to correctly manage them.

## Introduction

Systemic therapy for metastatic melanoma (MM) has dramatically changed in the past decades. Specific BRAF/MEK inhibitors for BRAF-mutant MM induce response rates of 70-80%, with progression-free survival (PFS) of 11-15 months and median overall survival (OS) of more than 2 years (1). Immune checkpoint inhibitors (ICIs), including monoclonal antibodies against CTLA-4 (cytotoxic T lymphocyte antigen-4), and PD-1 (programmed death antigen 1) or its ligand PD-L1, have induced durable response rates of about 15% and 40%, respectively (2, 3). Interestingly, when ipilimumab (anti-CTLA-4) is given in association with nivolumab (anti-PD-1), response rates rise to 60%. Unfortunately, at the same time, the incidence of immune-related adverse events (irAEs) increases up to 60%-85%, with the most affected organs being the skin, endocrine glands, gastrointestinal tract, lungs, liver, and kidney (4, 5).

Desmoplastic melanoma (DM) accounts for less than 4% of all melanomas. It is characterized by the presence of spindle-shaped melanocytes dispersed within dense collagenous stroma and scattered lymphoid aggregates. DM association with ultraviolet light-induced DNA damage makes it rich in neoantigens and particularly suitable for ICIs treatment. However, strong ICIs-induced anti-tumor immune responses can expose patients to a higher risk of developing irAEs (6–10). Whether the magnitude of irAEs is correlated with better clinical outcomes in melanoma and other neoplasms is still a matter of debate (1, 4, 5, 11, 12).

Here, we report the case of a patient with metastatic DM successfully treated with a combination of anti-CTLA-4 and anti-PD-1, who developed numerous serious immune-related side effects.

## Case presentation

### Clinical history

In February 2022, a 53-year-old man came to our Unit of Rare Tumors and Melanoma, at Istituto Tumori of Bari, Italy.

The patient’s clinical history began in October 2021 when he underwent excision of a left supra-axillary skin nodulation and an incisional biopsy of another larger lesion in the same area. Histopathological diagnosis was suggestive of malignant peripheral nerve sheath neoplasia (MPNST) of superficial soft tissues.

In December 2021, CT staging scan showed increased skin thickness at the left scapular site, with nodulations in the subcutaneous adipose tissue and lung nodules at the left upper lobe (5 mm), left lower lobe (25 mm), and the middle lobe lateral segment (3 mm). PET-FDG performed in January 2022 confirmed cutaneous-subcutaneous lesions at the left suprascapular region (SUV 28) and pulmonary lesions at the upper segment of the left lower lobe (SUV 14).

**Figure 1:**
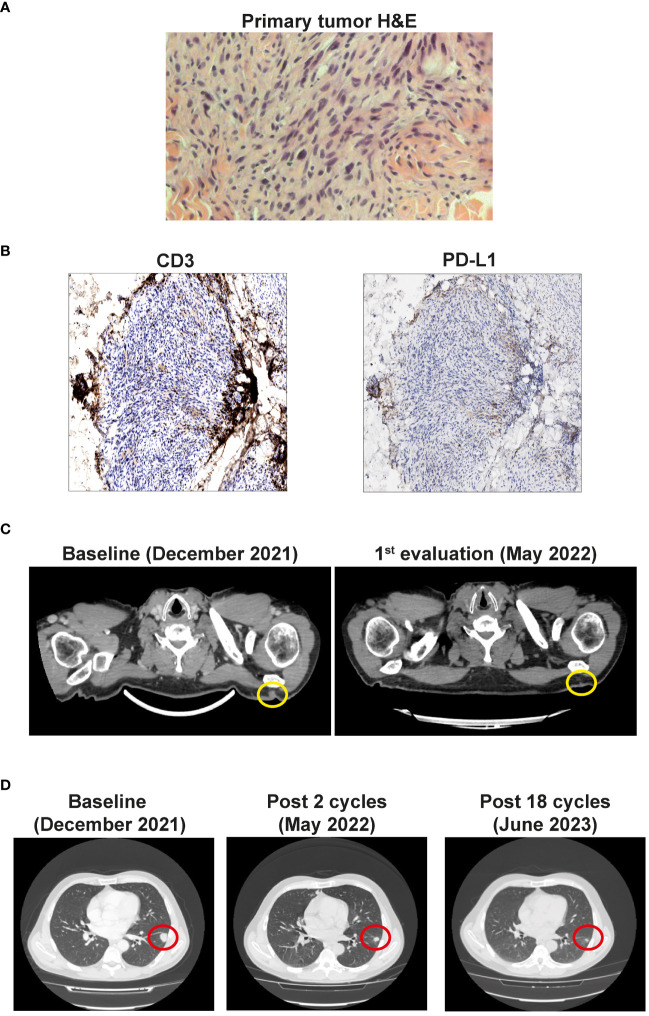
**(A)** Primary tumor histology. Hematoxilyn-eosin (H&E) staining of the primary tumor. **(B)** Left panel: Non-brisk T cell infiltration (anti-CD3 immunohistochemistry). Right panel: Negative tumor PD-L1 expression. Magnification 20x. **(C)** CT scan showing the cutaneous and subcutaneous lesions (yellow circles) before the start of the therapy (Baseline) and after 4 cycles (1st evaluation). **(D)** CT scan of the lung metastases (red circles) before the start of the therapy (Baseline), after 3 cycles, and after 18 cycles of immunotherapy.

In February 2022, after histological review at our Institute, the diagnosis of MPNST was updated to desmoplastic melanoma with Breslow thickness of 5 mm, Clark level V, mitosis 1x10Hpf, and absent ulceration (**Figure 1A**). PD-L1 expression was absent on tumor cells and TIL (Tumor infiltrating lymphocytes) were mostly excluded and localized in the peritumoral area (**Figure 1B**). Molecular analysis performed using next-generation sequencing (NGS) with a panel of 324 genes and introns of 36 genes involved in rearrangements, showed wild-type BRAF and pathological mutations in different genes (**Table 1**). The high tumor mutational burden and mutations in NF1, TERT, TP53, and NOTCH3 genes were concordant with the UV signature (10).

**Table 1:** Genomic profile of the primary DM tumor.

*Tumor Mutational Burden*	229 Muts/Mb
*Microsatellite status*	MS - Stable
**Gene Alterations**	**Variant allelic frequency (VAF)**
*APC* (T829fs*13) c.2486_2487del	33.3%
*NF1* (R2517*) c.7549C>T	53.6%
*TSC2* (R505*) c.1513C>T	39.9%
*ERBB4* (E563K) c.1687G>A	31.3%
*FLT1* (R281Q) c.842G>A	31.9%
*KDM5A amplification*	amplification - equivocal
*KDR* (G494E) c.1481G>A	53.5%
*NOTCH3* (3327 + 1G>A)	23.5%
*PBRM1* (Q779*)	53.4%
*RAF1* (S259F) c.776C>T	35.5%
*TERT* (-146C>T)	47.5%
*TP53* (P278F) c.832_833delCCinsTT	58.9%

### Treatment and response

In light of DM clinical aggressiveness and the lack of PD-L1 expression, the patient was treated with ipilimumab 3 mg/kg and nivolumab 1 mg/kg intravenously for 4 cycles, followed by nivolumab alone at the flat dose of 480 mg every 4 weeks

After two cycles, clinical complete remission of the left scapular lesions was observed. CT scan performed in May 2022, after 4 cycles of combined therapy, confirmed the complete regression of the cutaneous and subcutaneous lesions, and a partial response of lung metastases (**Figures 1C, D**).

**Figure 2:**
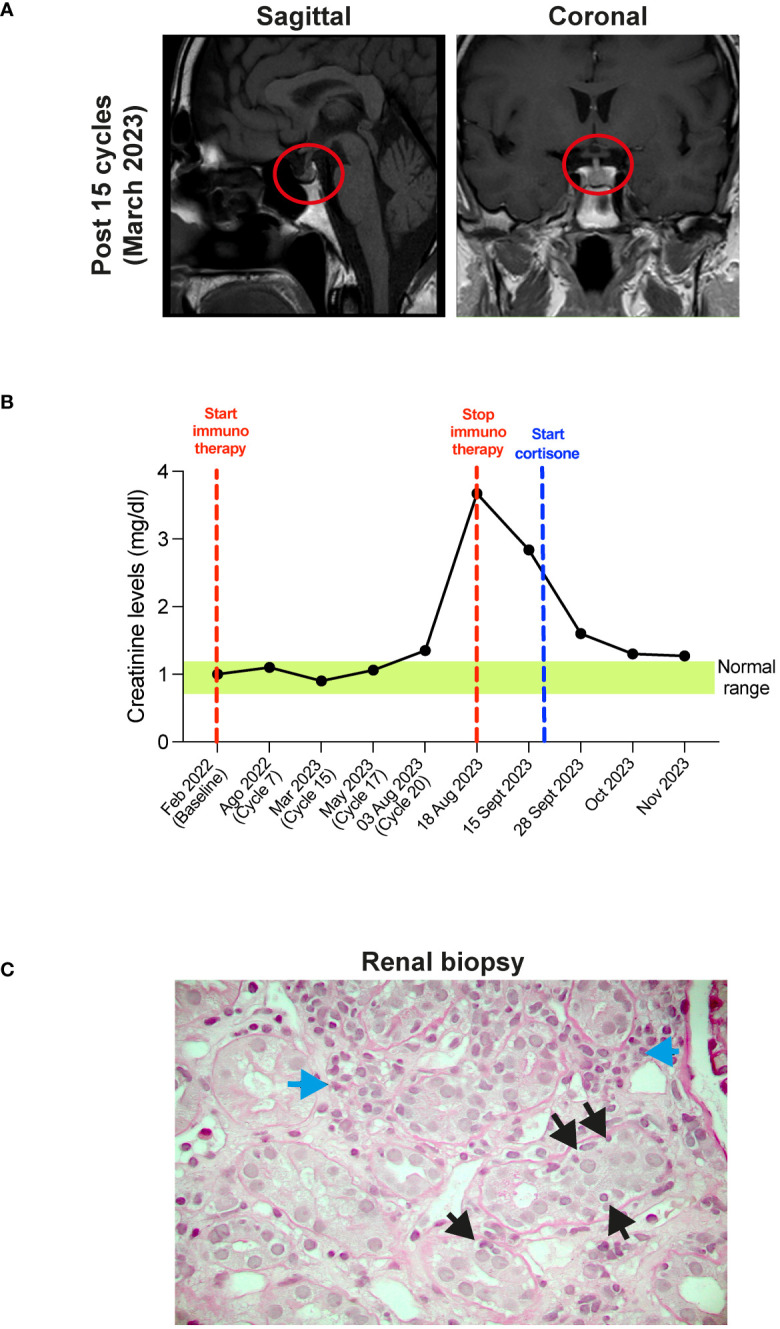
**(A)** MRI of the brain after 5 cycles of therapy. Sagittal (left panel) and coronal (right panel) planes show the thickening of the pituitary gland (red circles). **(B)** Timeline of the creatinine plasma levels (mg/dl). Red dotted lines show start and stop of the immunotherapy, blue dotted line show start of cortisone treatment. Green box shows the normal creatinine plasma levels. **(C)** Histopathological features of acute interstitial nephritis. Blue arrows show interstitial lymphomonocytic infiltrate, yellow arrows show tubulitis (Periodic Acid Schiff x400).

At the same time, the patient exhibited hyposthenia G2 and headache G1. Blood tests documented adrenal deficiency with a low value of cortisol 9.7 ng/ml (normal range 57-194) and ACTH of 2.0 pg/ml (normal range 5-63), associated with subclinical hypothyroidism with TSH 0.08 µUI/ml (normal range 0.25-5.0), fT3 5.18 pg/ml (2.0-5.0), fT4 1.75 ng/dl (0.7-1.7). Brain MRI showed a slight thinning of the pituitary gland (**Figure 2A**). Therefore, replacement therapy with thyroxine and cortone acetate was started (hydrocortisone, 37.5 mg cps/day), while continuing nivolumab immunotherapy.

CT re-evaluation scans, performed in September 2022, January 2023, and June 2023 confirmed the persistence of a complete response in the left scapular lesion, and a partial response of pulmonary lesions (**Figure 1D**). In August 2023, after 20 cycles of therapy, the patient complained of slight fever and asthenia with creatinine levels of 3.67 mg/dl (0.67 - 1.17) (**Figure 2B**). At this stage, immunotherapy was suspended, and patient was referred to a nephrological consultation. Kidney biopsy evidenced an acute interstitial nephritis (**Figure 2C**). Intravenous corticosteroid therapy with 1 mg/kg methylprednisolone was promptly started, and it induced a rapid improvement of renal function and creatinine normalization after one week. The following week, corticosteroid was switched to the oral formulation and progressively reduced (prednisone 37.5 mg/day for one month, followed by 25 mg/day for another month, followed by 12.5 mg/day) (**Figure 2B**). Currently, the patient is in close follow-up with medical examination, blood and urine analysis, and PET-FDG performed every 4 months. He has normal renal functionality and no disease relapse after 26 months from starting immunotherapy and after 9 months from its definitive suspension.

## Discussion

Desmoplastic melanoma (DM) is a rare subtype of melanoma characterized by high immunogenicity due to its association with ultraviolet light DNA damage, which makes it particularly suitable for immune checkpoint inhibitors (ICIs) treatment. Our patient showed a high tumor mutational burden and mutations in NF1, TERT, TP53, and NOTCH3 genes that confirm the UV-specific signature of DM (6, 10). He had a rapid and deep response to anti-CTLA-4/anti-PD-1 immunotherapy, despite the lack of PD-L1 expression, notoriously associated with a worse response to ICIs (4, 5). However, the patient developed serious immune-related adverse events (irAEs) including hypothyroidism, adrenal deficiency, and acute interstitial nephritis that led to the definitive suspension of treatment.

Different reports have shown that the incidence of irAEs in patients receiving ICIs can be as high as 60%–85%, depending on the use of mono- or combination immunotherapy. The most affected organs include skin, endocrine glands, gastrointestinal tract, lungs, and liver. Kidney toxicity is less common, but the incidence is rising as therapy with these agents continues to increase (4, 5).

The close association between tumor immunogenicity (mutational burden, baseline tumor-specific neoantigens, and CD8 T-cell Infiltration) and irAE during ICI therapies has been reported by several authors (13, 14). Originally conceived to selectively stimulate anti-tumor T cells (15), anti-CTLA-4 monoclonal antibodies have been shown to induce pan-T cell activation in clinical settings, compromising the host's immune tolerance to healthy self-tissues. As a result, autoimmune reactions have emerged as the nemesis of cancer immunotherapy (16).

To mitigate the irAEs arising from an iatrogenic auto-GVHD reaction (17), an ultra-low-dose ICI protocol has been developed. In a retrospective analysis of 131 unselected stage IV solid cancer patients with 23 different histological types who exhausted all conventional treatments, ultra-low-doses of ipilimumab (0.3 mg/kg) plus nivolumab (0.5 mg/kg) combined with hyperthermia and interleukin-2, resulted significantly safer than the registered protocol doses, without compromising efficacy (17). These data suggest that ultra-low doses may be not only safer but also cheaper than registered doses. Patil et al. reported results from a randomized clinical trial showing a significant and clinically meaningful benefit from incorporating ultra-low dose nivolumab (20 mg flat dose once every 3 weeks) into triple metronomic therapy (methotrexate 9 mg/m2 once a week, celecoxib 200 mg twice daily, and erlotinib 150 mg once daily) to treat patients with advanced head and neck cancer. This treatment regimen dramatically reduced the financial cost of immunotherapy, with the potential to increase access and improve patient outcomes in low- and middle-income countries (18). However, several points remain to be clarified, for example (*i*) whether ultra-low doses of ICIs are equivalent to the currently approved doses when administered as monotherapy, and (*ii*) if the results obtained so far can be extended to all types of cancer and patient populations (19).

It is still debated whether there is a direct correlation between ICIs effectiveness and the degree of treatment-induced toxicity (4, 5, 20). A landmark analysis in patients with advanced melanoma showed that the efficacy of pembrolizumab was not affected by the occurrence of irAEs or systemic corticosteroid use (1). Other reports showed that irAEs are strongly correlated with better survival and higher response rates in patients with melanoma (11), advanced gastric cancer (12) and NSCLC receiving anti-PD-1 therapy (21, 22).

Limited literature exists on the incidence, time of onset, and risk factors for multiorgan systems irAEs, which occurred in about 5% of ICI- treated patients. Combination therapy (anti-CTLA-4 plus anti-PD-1/PD-L1) is associated with an increased risk of multiorgan systems irAEs. Interestingly, severe sequential irAEs involving multiple organs are often associated with a durable complete response despite early therapy discontinuation (23).

No prognostic factors have so far been associated with multiorgan irAEs. A study in patients with non-small cell lung cancer showed a correlation between atezolizumab-induced irAEs and good performance status, lower baseline neutrophil-lymphocyte ratio, and good or intermediate lung immune prognostic index score (24). Future trials should consider routine reporting of data on multiorgan toxicities in addition to organ-specific toxicities.

As mentioned above, a significant percentage of patients treated with ICIs also present endocrine irAEs. Combination therapy (anti-CTLA-4 plus anti-PD-1/PD-L1) is associated with an increased risk and prevalence of endocrine irAEs (25). Hypophysitis and thyroid dysfunctions are the most common endocrine irAEs, while cases of Type 1 diabetes mellitus and adrenal insufficiency are rarer. Most of the patients normally recover from pituitary-thyroid and pituitary-gonadal axis dysfunctions, while improvement of the pituitary-adrenal axis has been observed only in a few cases (25).

The incidence of acute kidney damage has been reported in 2% - 5% of patients treated with ICIs, and acute interstitial nephritis (AIN) is the predominant pathological sign (26). These numbers could be underestimated as many patients do not undergo kidney biopsy in the presence of mild renal toxicity. In addition, AIN could be masked by the steroid therapy prescribed for other irAEs. AIN is classically described as the triad of fever, rash, and eosinophilia in association with elevated serum creatinine, but these factors are present in 5%–10% of the cases. Classically, the onset of acute kidney injury ranges between 2 to 11 months from the start of ICIs therapy. In our patient AIN arose after more than 18 months of therapy, showing that renal toxicity could occur later. Histologically, AIN is characterized by the presence of inflammatory infiltrates and edema in the kidney interstitium (27). Creatinine, electrolytes, and urinalysis tests before, and during each cycle of ICIs treatment are crucial for an early identification of kidney toxicity. Nevertheless, a kidney biopsy would be needed to better understand the etiopathogenesis and the degree of damage of renal dysfunction. After adequate corticosteroid treatment, most of the patients recover their kidney function, with about 10% of them progressing to chronic kidney disease (26, 27). For this reason, the prompt administration of high doses of steroids (0.8-1 mg/kg) with a slow taper is recommended. In our patient, the administration of 1 mg/kg methylprednisolone induced a rapid recovery of renal dysfunction after 1 week until complete normalization.

Whether or not immunological therapy should be definitively discontinued in case of severe renal toxicity is still an open question. An eventual ICIs discontinuation would depend on several factors, such as (i) the state of the disease at the time of withdrawal, (ii) the type of response to ICIs, (iii) the duration of therapy already administered, (iv) the availability of other therapeutic options, (v) the patient’s will (28, 29). In case of relapse, ICIs rechallenge after recovery from kidney toxicity could be considered, potentially with the concomitant use of low doses of steroids to reduce the risk of kidney toxicity recurrence. Considering the complete response to ICIs treatment and the numerous irAEs, we decided to stop immunotherapy and keep our patient in close follow-up including medical examination, blood, and urine analysis and PET-FDG performed every 4 months. After 26 months from starting ICI immunotherapy and after 9 months from its definitive suspension, the patient has normal renal functionality, and no disease relapse has been documented.

In conclusion, our case has demonstrated that ICIs treatment is highly effective in DM, but ICIs-related toxicity could represent the price to pay to achieve disease remission. Therefore, clinicians should closely monitor DM patients during ICIs therapy for severe irAEs occurrence to properly identify and treat them.

## Data availability statement

The raw data supporting the conclusions of this article will be made available by the authors, without undue reservation.

## Ethics statement

Written informed consent was obtained from the individual(s) for the publication of any potentially identifiable images or data included in this article.

## Author contributions

TS: Data curation, Formal analysis, Methodology, Validation, Writing – original draft, Writing – review & editing. RV: Data curation, Formal analysis, Methodology, Writing – original draft, Writing – review & editing. BA: Writing – review & editing. LF: Methodology, Writing – review & editing. MZ: Methodology, Writing – review & editing. MR: Methodology, Writing – review & editing. RP: Methodology, Writing – review & editing. ST: Methodology, Writing – review & editing. ID: Data curation, Methodology, Writing – review & editing. SS: Conceptualization, Data curation, Methodology, Writing – review & editing. MG: Conceptualization, Data curation, Supervision, Writing – original draft, Writing – review & editing.
